# Biosystematics studies on *Elymus breviaristatus* and *Elymus sinosubmuticus* (Poaceae: Triticeae)

**DOI:** 10.1186/s12870-022-03441-y

**Published:** 2022-02-01

**Authors:** Lu Tan, Qing-Xiang Huang, Yang Song, Dan-Dan Wu, Yi-Ran Cheng, Chang-Bin Zhang, Li-Na Sha, Xing Fan, Hou-Yang Kang, Yi Wang, Hai-Qin Zhang, Yong-Hong Zhou

**Affiliations:** 1 Triticeae Research Institute, Sichuan Agricultural University, Wenjiang 611130, Chengdu, Sichuan China; 2 State Key Laboratory of Crop Genetic Exploration and Utilization in Southwest China, Sichuan Agricultural University, Wenjiang 611130, Chengdu, Sichuan China; 3 Sichuan Academy of Grassland Science, Chengdu, 610000 Sichuan China; 4 College of Grassland Science and Technology, Sichuan Agricultural University, Chengdu, 611130 Sichuan China

**Keywords:** *Campeiostachys*, Chromosome pairing, Genomic in situ hybridization, Reproduction isolation, Biosystematics

## Abstract

**Background:**

*Elymus breviaristatus* and *Elymus sinosubmuticus* are perennial herbs, not only morphologically similar but also sympatric distribution. The genome composition of *E. sinosubmuticus* has not been reported, and the relationship between *E. sinosubmuticus* and *E. breviaristatus* is still controversial. We performed artificial hybridization, genomic in situ hybridization, and phylogenetic analyses to clarify whether the two taxa were the same species.

**Results:**

The high frequency bivalent (with an average of 20.62 bivalents per cell) at metaphase I of pollen mother cells of the artificial hybrids of *E. breviaristatus* (**StYH**) × *E. sinosubmuticus* was observed. It illustrated that *E. sinosubmuticus* was closely related to *E. breviaristatus*. Based on genomic in situ hybridization results, we confirmed that *E. sinosubmuticus* was an allohexaploid, and the genomic constitution was **StYH**. Phylogenetic analysis results also supported that this species contained **St**, **Y**, and **H** genomes. In their F_1_ hybrids, pollen activity was 53.90%, and the seed setting rate was 22.46%. Those indicated that the relationship between *E. sinosubmuticus* and *E. breviaristatus* is intersubspecific rather than interspecific, and it is reasonable to treated *E. sinosubmuticus* as the subspecies of *E. breviaristatus*.

**Conclusions:**

In all, the genomic constitutions of *E. sinosubmuticus* and *E. breviaristatus* were **StYH**, and they are species in the genus *Campeiostachys.* Because *E. breviaristatus* was treated as *Campeistachys breviaristata*, *Elymus sinosubmuticus* should be renamed *Campeiostachys breviaristata* (Keng) Y. H. Zhou, H. Q. Zhang et C. R. Yang subsp. *sinosubmuticus* (S. L. Chen) Y. H. Zhou, H. Q. Zhang et L. Tan.

**Supplementary Information:**

The online version contains supplementary material available at 10.1186/s12870-022-03441-y.

## Background

The tribe Triticeae includes about 450 species, of which about 75% are polyploid [1, 2]. Since Löve [1] proposed that the species with the same genome or same genome combinations were classified into one genus, about 30 genera were recognized by most of the grass scientists [3–9]. *Elymus* sensu lato (*Elymus* s.l.) is the largest genus of Triticeae, and it contains seven basic genomes: **St**, **H**, **P**, **W**, **Ns**, **Y**, and **Xm** [3, 8, 10–12]. **St** genome is from *Pseudoroegneria* (Nevski) Löve, **H** genome is from *Hordeum* L., **P** genome is from *Agropyron* Gartn., **W** genome is from *Australopyrum* (Tzvelev) Löve, **Ns** genome is from *Leymus* Hochst. The origin of **Y** and **Xm** is still unknown [3, 7, 9, 13]. Based on the genome combinations, *Elymus* s.l. was further divided into ten genera, including *Elymus* sensu stricto (*Elymus* s.s.) (**StH**), *Roegneria* C.Koch (**StY**), *Hystrix* Moench (**StH**/**NsXm**), *Stenostachys* Turcz. (**HW**), *Douglasdeweya* C.Yen, J.L.Yang et B.R.Baum (**StP**), *Kengyilia* C.Yen et J.L.Yang (**StYP**), *Campeiostachys* Drobov (**StYH**), *Anthosachne* Steudel (**StYW**), *Pascopyrum* Á. Löve (**StHNsXm**), and *Connorochloa* Barkworth, S.W.L.Jacobs et H.Q.Zhang (**StYWH**) [5–8, 13–16]. Of which, due to the dominant effect of the genes of the **St** and **H** genomes, it is challenging to distinguish *Campeiostachys* from *Elymus* s.s. based on single or combined morphological characters [6, 8, 17]. Moreover, the genome composition of many polyploid species in *Elymus* s.s. and *Campeiostachys* is still unknown, resulting in the classification of many species in these two genera remains controversial [6, 8]. Although the genome composition of some species is determined, their biosystematics remains controversial due to their similar morphological features.

*Elymus breviaristatus* (Keng) Keng ex Keng f. and *Elymus sinosubmuticus* S. L. Chen is sympatric species mainly distributed on hillsides in Sichuan, Qinghai, and Ningxia, China [9, 18–21]. Morphologically, those two species are quite similar, and the only difference exists in their awn length. *E. breviaristatus* has short awn (2–5 mm), while *E. sinosubmuticus* possesses degenerated awn only 0-2 mm in length [9, 19, 22]. Overlapping geographical distribution and similar morphology, whether or not they are the same species is under controversy. Based on the morphological characteristics and the results of RPDA analysis, these two species were treated as independent biological species [8, 9, 19, 21, 23]. Zhang et al. [24] suggested that *E. breviaristatus* and *E. sinosubmuticus* were the same species by comparing the leaf anatomical characteristics.

The chromosome pairing behavior of hybrid F_1_ at meiosis metaphase can be used to indicate chromosome homology and evolutionary relationship between genus or species in Triticeae [25, 26]. Genomic in situ hybridization (GISH) can effectively examine the genome composition and chromosomal rearrangement of polyploid species [27–32]. Cytologically, *E. breviaristatus* and *E. sinosubmuticus* are allohexaploid (2n = 6x = 42) perennial wheatgrass [1, 8, 21, 33], but Mason-Gamer et al. [34] reported that *E. breviaristatus* is tetraploid with **StH** genome. Based on the genome analysis and GISH, Yang et al. [35] recognized that *E. breviaristatus* was a hexaploid with the **StYH** genome and treated it as *Campeistachys breviaristata* (Keng) Y.H.Zhou, H.Q.Zhang et C.R.Yang. However, the genome composition of *E. sinosubmuticus* has not been reported at present.

Cytological and phylogenetic analyses are practical tools to determine the genome composition and explore the interspecies and intergeneric relationships of the species in Triticeae [36–39]. Molecular phylogeny analysis based on the single- or low-copy nuclear genes is less susceptible to concerted evolution and can be a handy marker for polyploid phylogeny [40–44]. Furthermore, Petersen et al. [43] found a correspondence between DNA sequences of diploid donors and allopolyploids in Triticeae. Therefore, more and more single-copy nuclear genes have been used to determine the genome composition and phylogenetic relationship of Triticeae. *Acc1* and *DMC1* sequences have higher evolutionary rates and have been widely applied in the phylogenetic study of the genera of Triticeae, such as *Triticum*, *Kengyilia*, *Leymus*, *Roegneria*, *Hystrix*, etc. [44–49]. In the present study, GISH, single-copy nuclear genes, and artificial hybridization were used to investigate the genome composition of *E. sinosubmuticus* and explore the biosystematics relationships between *E. breviaristatus* and *E. sinosubmuticus*.

## Results

### Meiosis and fertility of parentals and F_1_ hybrids

Five hybrids were obtained from the combination of *E. breviaristatus* × *E. sinosubmuticus*. We observed the chromosome pairing of PMCs at metaphase I (MI) of parents and hybrids (Table 1). Meiosis of *E. breviaristatus* and *E. sinosubmuticus* forming mostly ring bivalents, with an average of 21.00 and 20.92 bivalents per cell, respectively (Table 1; Fig. S1, see Additional file 1). The F_1_ hybrids of *E. breviaristatus* × *E. sinosubmuticus* was a hexaploid (2n = 42), showing an average of 0.50 univalents, 20.62 bivalents, 0.06 trivalent, and 0.02 quadrivalents (Table 1; Fig. S1, see Additional file 1). The chiasmata per cell were 37.70, with a c-value of 0.89, suggesting that they were genetically affinity species and had similar **StYH** genome constitution.

**Table 1:** Meiotic associations at metaphase I in pollen mother cells of parental species and their hybrids

Species or hybrids	2n	No. of cells observed	Chromosome association						Chiasmata/cell	c-value
			I	II (Total)	II (Ring)	II (Rod)	III	IV		
*Elymus breviaristatus*	42	50	-	21.00	20.74	0.26	-	-	41.74	0.99
			-	(21)	(19–21)	(0–2)	-	-		
*Elymus sinosubmuticus*	42	50	0.08	20.92	20.72	0.20	-	-	41.64	0.99
			(0–2)	(20–21)	(19–21)	(0–2)	-	-		
*E. breviaristatus* × *E. sinosubmuticus*	42	50	0.50	20.62	16.88	3.74	0.06	0.02	37.70	0.89
			(0–2)	(19–21)	(13–19)	(1–8)	(0–1)	(0–1)		

Pollen grains of parents (*E. breviaristatus* and *E. sinosubmuticus*) showed a high level of stainability, was 92.91% and 92.32%, respectively. The percentage of stained pollen grains of the hybrids was comparatively high at 53.90%. The seed setting rate of *E. breviaristatus* and *E. sinosubmuticus* were 89% and 87%, respectively. And the seed setting rate of their hybrids was 22.46%, indicating that the two species were highly affinities.

### GISH analysis

**Fig. 1:**
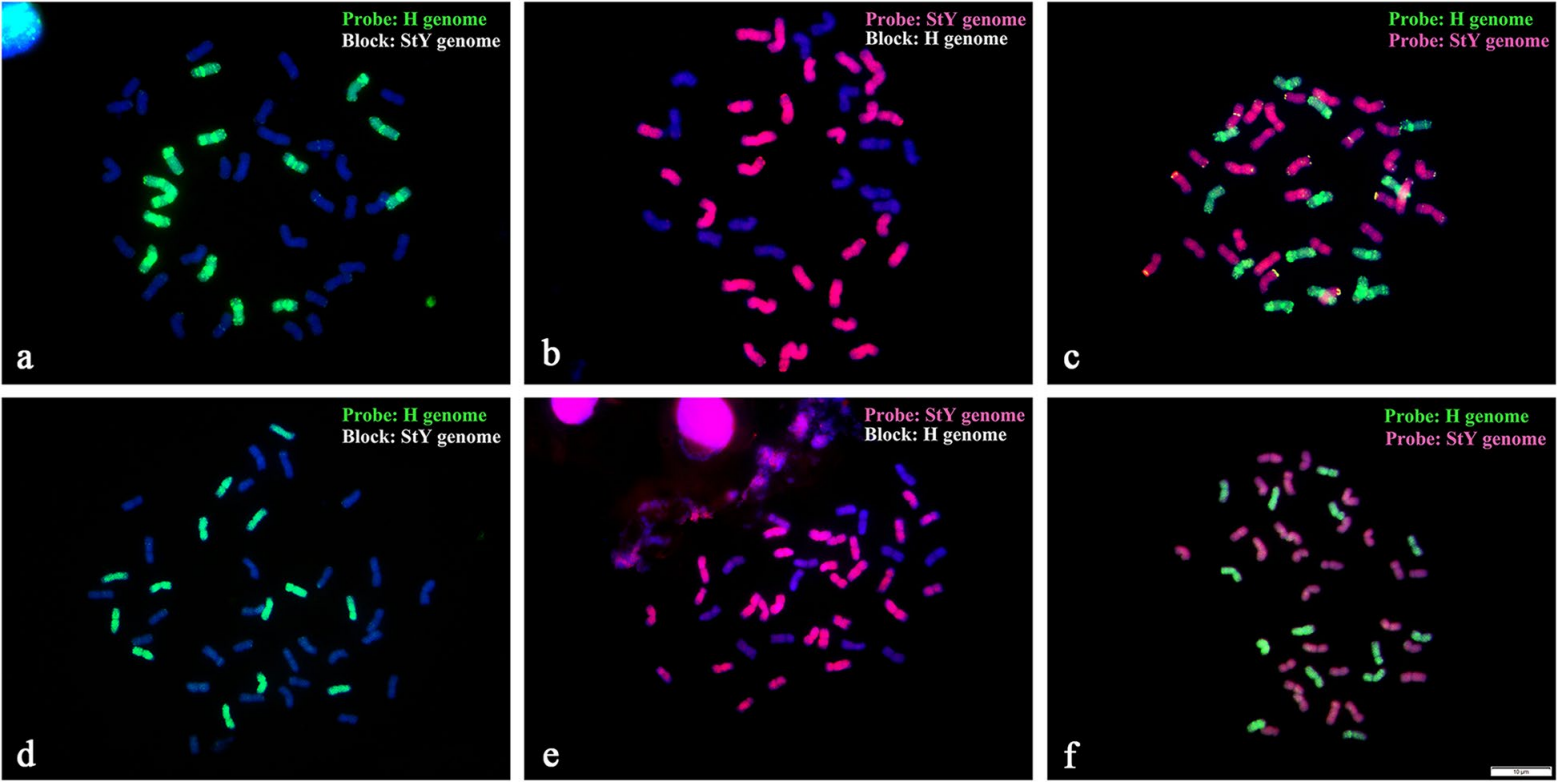
GISH on somatic metaphase cells from root tips of *Elymus sinosubmuticus* and *Elymus breviaristatus*. **a-c**, *E. sinosubmuticus*. **a**, 14 chromosomes showed **H** genome single which from *Hordeum bogdanii* when blocked with **StY** genome which from *Roegneria ciliaris*. **b**, 28 chromosomes showed **StY** singles when blocked with **H** genome. **c**, 14 chromosomes showed **H** genome singles and 28 chromosomes showed **StY** singles when **StY** genome and **H** genome as probes. **d-f**, *E. breviaristatus*. **d**, 14 chromosomes showed **H** genome singles when blocked with **StY** genome. **e**, 28 chromosomes showed **StY** singles when blocked with **H** genome. **f**, 14 chromosomes showed **H** genome singles and 28 chromosomes showed **StY** singles when **StY** genome and **H** genome as probes. Scale bar equal 10 μm

To confirm the genome constitution of *E. sinosubmuticus*, root meristem cells that went through mitosis metaphase were collected for GISH. It showed that *E. sinosubmuticus* is a hexaploid with 42 chromosomes. Of which, 28 chromosomes were hybridized with the **StY** probe (from *Roegneria ciliaris* (Trin.) Nevski) when blocked by the **H** genome (from *Hordeum bogdanii* Wilensky) (Fig. 1a). And 14 chromosomes were hybridized with the **H** probe when blocked by the **StY** genome (Fig. 1b). Double-color GISH showed that 28 chromosomes were stained by the **StY** probe (in red), and 14 chromosomes were labeled by the **H** probe (in green) (Fig. 1c). In accordance, *E. breviaristatus* also contains 42 chromosomes, and 28 chromosomes displayed **StY** signals on the entire arm, and 14 chromosomes showed **H** signals (Fig. 1d, e, f).

### Phylogenetic analyses

**Fig. 2:**
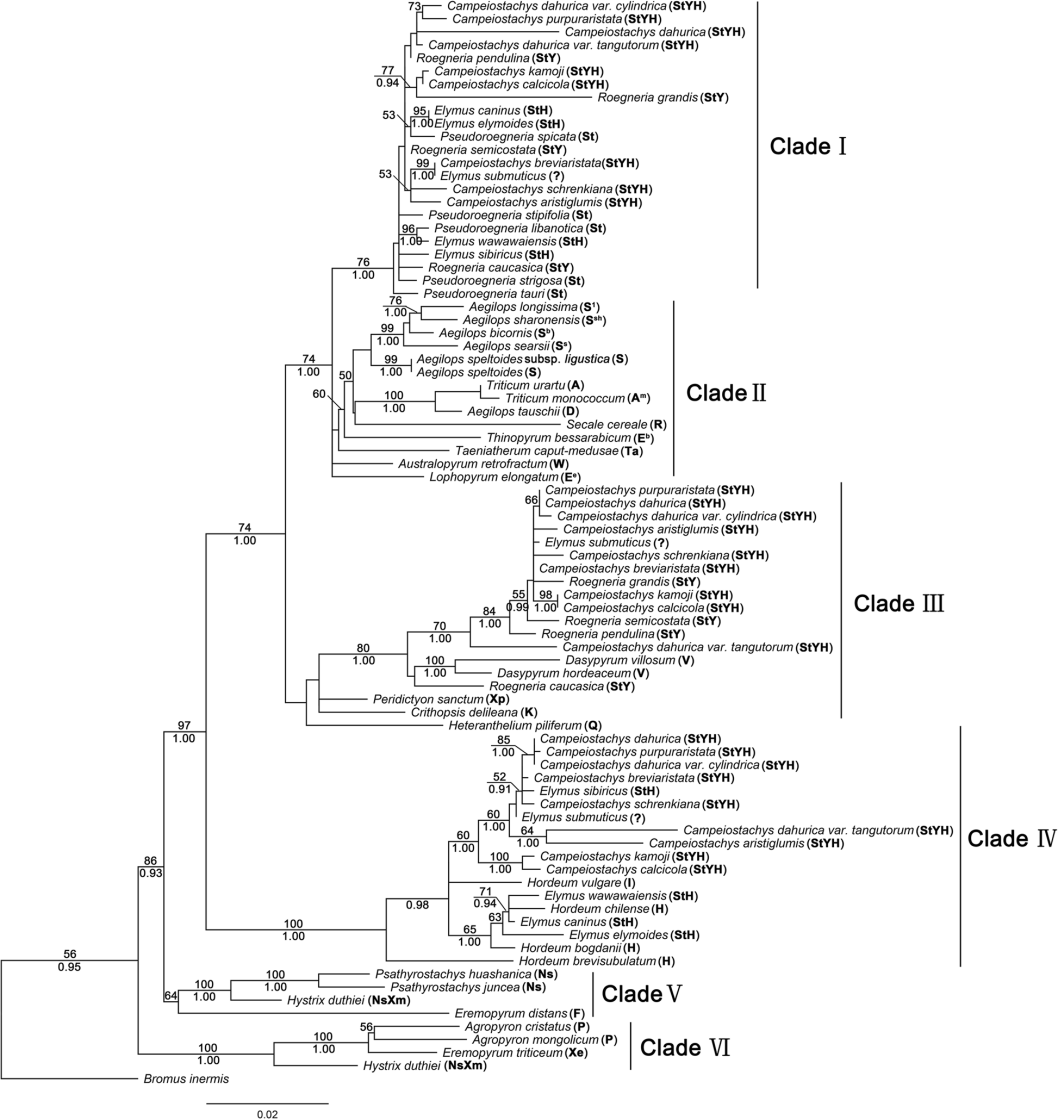
Maximum likelihood tree derived from *Acc1* sequences data. The bold indicated sequences from *Elymus sinosubmuticus* and *Elymus breviaristatus*. The capital letters in brackets after the species name indicate the genome composition of the species, and the “?” indicate the genome composition of the species is unknown. The numbers above and below the branches indicate bootstrap values > 50% and Bayesian posterior probability values > 90%, respectively

The Acetyl-CoA carboxylase (*Acc1*) sequences length of *E. sinosubmuticus* ranged from 1421 to 1443 bp, and *E. breviaristatus* went from 1428 to 1441 bp. The *Acc1* data matrix of sequences was analyzed based on maximum likelihood (ML) using the model TIM1 + I + G (-Ln likelihood = 8147.4309). The assumed nucleotide frequencies were A = 0.2555, C = 0.1794, G = 0.2116, T = 0.3535. The tree generated by Bayesian analysis was similar to ML analysis. All the *Acc1* sequences were grouped into six clades (Fig. 2). The sequence from *E. breviaristatus* and *E. sinosubmuticus* were divided into clade I, clade III, and clade IV, respectively. Clade I contained the *Pseudoroegneria*, *Elymus*, *Roegneria*, and *Campeiostachys* species (BS = 100%, PP = 76%,). Clade III included in the *Dasypyrum*, *Roegneria*, and *Campeiostachys* species (BS = 100%, PP = 80%,). Clade IV grouped with the *Hordeum*, *Elymus*, and *Campeiostachys* species (BS = 100%, PP = 100%).

**Fig. 3:**
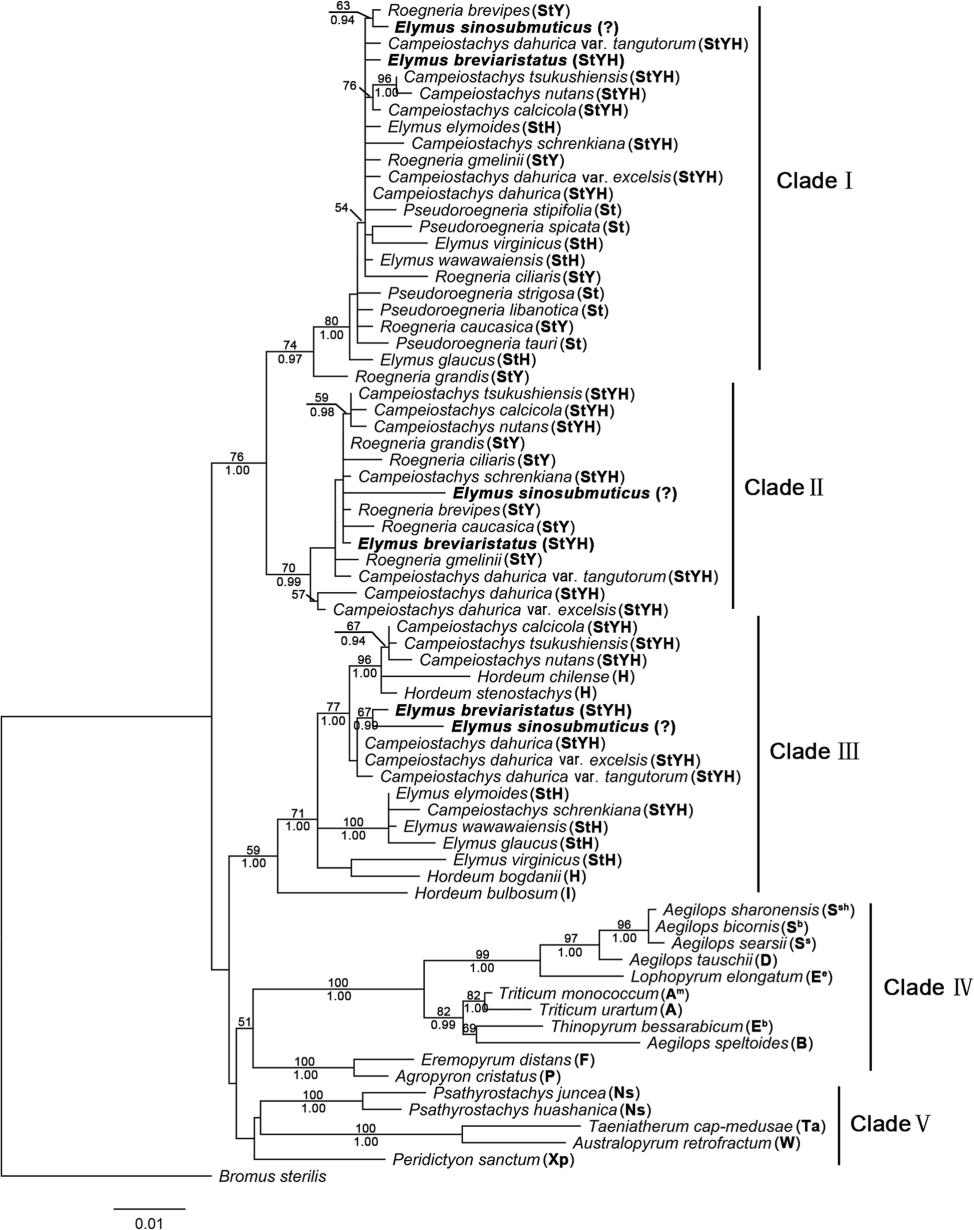
Maximum likelihood tree derived from *DMC1* sequences data. The bold indicated sequences from *Elymus sinosubmuticus* and *Elymus breviaristatus*. The capital letters in brackets after the species name indicate the genome composition of the species, and the “?” indicate the genome composition of the species is unknown. The numbers above and below the branches indicate bootstrap values > 50% and Bayesian posterior probability values > 90%, respectively

A total of 71 disrupted meiotic cDNA (*DMC1*) sequences were used for ML analysis, *Bromus sterilis* as the outgroup. TPM2uf + G as the best-fit model, -Ln likelihood = 5355.5355. The assumed nucleotide frequencies were A = 0.2576, C = 0.2120, G = 0.2085, T = 0.3220. The tree generated by Bayesian analysis was similar to ML analysis. The *DMC1* sequences from *E. breviaristatus* and *E. sinosubmuticus* were divided into three clades (Fig. 3). In clade I, grouped with the diploid species (*Pseudoroegneria*), and tetraploid species (*Elymus* and *Roegneria*), and hexaploid species (*Campeiostachys*) (BS = 97%; PP = 74%). In clade II, their sequences grouped with the species of the genus *Roegneria* and *Campeiostachys* (BS = 99%, PP = 70%). In clade III, grouped with the diploid species (*Hordeum*) and the species of the genus *Elymus* and *Campeiostachys* (BS = 100%, PP = 59%). Clade IV and clade V grouped with the other diploid species in Triticeae (Fig. 3).

## Discussion

### *Elymus sinosubmuticus* contains StStYYHH genome

In this study, genome analysis, GISH, and phylogenetic analyses indicate that *E. sinosubmuticus* is an allohexaploid with the **StYH** genome. Based on the genome combinations, the species with **St**, **Y**, **H** genomes should be classified into the genus of *Campeiostachys* [6]. Previously, *E. sinosubmuticus* was classified into the *Elymus* genus based on morphological traits and geographic distribution [8, 9, 21]. Phenotype is the co-consequence of genetics and environments. Some studies have shown that there are cryptic species (such as *Roegneria panormitana* (Parl.) Nevski and *R. heterophylla* (Bornm. ex Melderis) C. Yen, J. L. Yang and B. R. Baum) and cryptic genera (such as *Elymus* and *Campeiostachys*) in Triticeae [6, 8, 50]. The former has complete reproductive isolation, and the latter has different genome combinations. None of them can be distinguished morphologically. Therefore, for the Triticeae, especially for cryptic genera, it is not accurate to classify the species based only on morphological traits. Our study is reasonable to classify *E. sinosubmuticus* into the genus *Campeiostachys* based on the genome analysis, GISH, and phylogenetic analyses results*.*

### Biosystematics relationships of *E. breviaristatus* and *E. sinosubmuticus*

There is still debate whether or not *E. breviaristatus* and *E. sinosubmuticus* are the same species [8, 23]. Karyotype analysis showed that those two hexaploid species belonged to type 2A [20]. From the leaf anatomical structure, the comparison of the leaf anatomical characteristics showed that the external morphology of *E. breviaristatus* and *E. sinosubmuticus* had little difference in leaf anatomy, and it was difficult to distinguish. Therefore, *E. breviaristatus* and *E. sinosubmuticus* were the same species, and *E. sinosubmuticus* should be a synonym for *E. breviaristatus* [24]. Conversely, Zhou et al. [23] based on the results of RPDA analysis, despite the close relationship between them, there was a certain degree of nucleotide sequence difference, and they were independent biological species. The morphological characteristics of *E. breviaristatus* and *E. sinosubmuticus* we observed were differing little. Both species are perennial tufted plants, culms erect. Leaf-sheaths glabrous, leaf-blades flat, margins ciliate. Spikes nodding or curved, with sparse remote spikelets, two spikelets on each rachis node, green or purple-tinged. Lemma is lanceolate and with five nerves. Palea is equal to lemma. Anthers yellow. The most significant difference is the length of the lemma awn, *E. sinosubmuticus* is only 0-2 mm, and *E. breviaristatus* is 2-6 mm. In addition, many types of interspecific variations were found in our field studies.

A high chromosome pairing frequency of hybrid F_1_ can indicate that the parents are closely related [51, 52]. A species represents an independent gene pool in the evolutionary system, and reproductive isolation is the only factor for forming such independent gene pools in organismal evolution [15]. Accordingly, reproductive isolation is the only standard for species identification. In our study, the hybrid F_1_ of *E. breviaristatus* and *E. sinosubmuticus* has a high-frequency bivalent at MI (mean value of 20.62), suggesting that the three genomes of *E. breviaristatus* and *E. sinosubmuticus* has high homology, and they are closely related. But the percentage of stained pollen grains of hybrids was 53.90%, and the seed setting rate was 22.46%. This suggests genetic differentiation between the two taxa, leading to a degree of reproductive isolation. Yang et al. [35] reported that *E. breviaristatus* was a hexaploid with the **StYH** genome and treated it as *Campeiostachys breviaristata* (Keng) Y. H. Zhou, H. Q. Zhang et C. R. Yang. Combined with morphological characteristics and the fertility of hybrids, *E. sinosubmuticus* should be classified into the genus *Campeiostachys* as the subspecies of *E. breviaristatus* and renominated as *Campeiostachys breviaristata* (Keng) Y. H. Zhou, H. Q. Zhang et C. R. Yang subsp. *sinosubmuticus* (S. L. Chen) Y. H. Zhou, H. Q. Zhang et L. Tan.

## Conclusions

*Elymus sinosubmuticus* is allohexaploid wheatgrass, and the genome composition is **StYH**. Its morphological characteristics are very similar to *E. breviaristatus.* Simultaneously, *E. sinosubmuticus* and *E. breviaristatus* have a degree of reproductive isolation, and it is reasonable to treat *E. sinosubmuticus* as the subspecies of *E. breviaristatus*. Because *E. breviaristatus* was treated as *Campeiostachys breviaristata* by Yang et al. [35], therefore*, E. sinosubmuticus* should be renamed as *Campeiostachys breviaristata* (Keng) Y. H. Zhou, H. Q. Zhang et C. R. Yang subsp. *sinosubmuticus* (S. L. Chen) Y. H. Zhou, H. Q. Zhang et L. Tan.

## Methods

### Plant materials

In our study, *Elymus breviaristatus* and *Elymus sinosubmuticus* were collected from the field in Sichuan Province, China, and numbered ZY 17,004 and ZY 17,008 respectively. No permissions were necessary to collect seed samples. Yonghong Zhou and Haiqin Zhang identified the two plant materials. They were used for artificial hybridization, and the materials and F_1_ hybrids were cultivated in the greenhouse at Hongyuan, Sichuan. The voucher specimens of *E. breviaristatus* and *E. sinosubmuticus* were deposited in the Herbarium of Triticeae Research Institute of Sichuan Agricultural University, China (SAUTI). Apart from *E. breviaristatus* and *E. sinosubmuticus*, diploid species and relative polyploid species with different genome combinations (**StY**, **StH**, **StYH**) in Triticeae were also applied for phylogenetic analyses. The basic information about these sequences is listed in Additional file 2: Table S1.

### Hybridization and meiotic analysis

The hybridization procedure is as follows: after 2–3 days of the emasculation of the female parent, repeat pollinations with the corresponding mature pollens of the male parent were carried out. The female parents were used a plastic bag to isolate the pollen throughout the whole process. In crossing combination, *E. breviaristatus* was used as the male parent when crossed with *E. sinosubmuticus*, and *E. sinosubmuticus* was used as the male when hybridized with *E. breviaristatus*. The chromosome pairing of pollen grains of hybrids and parents was examined after fixing by Carnoy's Fluid II for 24 h. The mean pairing frequency of hybrids and parents at MI is described by Kimber and Alonso [53]. Mature pollen of hybrids and parents were detected activity after staining with I_2_-IK solution.

### Chromosome preparation and GISH

The roots were collected from adult plants, treated with nitrous oxide for three hours, fixed with 90% glacial acetic acid for 5 min, and kept with 70% alcohol. The chromosome was prepared by drop methods [54]. Using the CTAB method [55] extracted the total genomic DNA from leaves. DNA was labeled using DIG-Nick Translation Kit (Roche, Indianapolis, IN, USA). The green probes were labeled with fluorescein-12-dUTP, and the red probes were labeled with Texas-red-5-dCTP using the nick translation method. Hybridization procedure, detection, and visualization are referred to Han et al. [56]. For monochromatic GISH, the concentration ratio of probe genomic DNA and non-labeled blocking genomic DNA was 1:120 (ng/uL). For double-color GISH, the concentration ratio of probes was 1:1 (ng/uL). Images of GISH were captured by Olympus BX61 fluorescence microscopy (Japan). At least ten metaphase cells for each species were observed. Adobe Photoshop CS6 was used to proceed with the images.

### Sequence amplification and phylogenetic analyses

The *Acc1* and *DMC1* sequences were amplified with primers listed in Table 2. All polymerase chain reactions were amplified in a 50 uL reaction mixture, containing 25 uL 1 × phanta mix buffer, 1 mM dNTP mix, 1 uL DNA polymerase (Vazyme, Nanjing, China), 10 uM of each primer, 200 ng of template DNA, and distilled deionized water to the final volume. Polymerase chain reaction (PCR) products were cloned into the 007VS vector (TSINGKE Biological Technology, Beijing, China). At least 15 random independent clones were selected for sequencing by Sangon Biological Engineering and Technology Service Ltd. (Shanghai, China). DNA sequences were confirmed through BLAST nucleotide alignment on NCBI database. The multiple sequences were aligned, and manual adjustments were made using the ClustalX [57]. jModelTest 3.0 [58] was used to determine appropriate DNA substitution models and gamma rate heterogeneity. Phylogenetic analyses were conducted using the maximum-likelihood method in PhyML 3.0 [59] and Bayesian inference (BI) in MrBayes version 3.1.2 [60]. Statistical support for nodes in ML analysis was estimated by using 1000 fast bootstrap replicates. Bootstrap support (BS) value < 50% and posterior probabilities (PP) value < 90% was not included in figures.

**Table 2:** The primers used in this study

Gene	Name of primer	Sequence of primer (5’–3’)	Profiles
Acc1	AccF1	CCCAATATTTATCATGAGACTTGCA	1 cycle: 5 min 95℃; 35 cycles: 30 s 95℃, 30 s 56℃, 2min30s 68℃; 1 cycle: 10 min 68℃
	AccF2	CAACATTTGAATGAAThCTCCACG	
DMC1	TDMC1e10F	TGCCAATTGCTGAGAGATTTG	1 cycle: 4 min 95℃; 35 cycles: 1 min 95℃, 1 min 52℃, 1 min 72℃; 1 cycle: 10 min 72℃
	TDMC1e15R	AGCCACCTGTTGTAATCTGG	

## Supplementary Information

**Additional file 1. **

**Additional file 2. **

## Data Availability

All sequences data from this study were deposited in National Center for Biotechnology Information (NCBI) and the accession number are MT749376, MT749377, MT749378, MT749380, MT749381, MT749382, MT820539, MT820540, MT820541, MT820545, MT820546, MT820549.

## Abbreviations

Acc1: Acetyl-CoA carboxylase;
BS: Bootstrap support;
BI: Bayesian inference;
DMC1: Disrupted meiotic cDNA;
GISH: Genomic in situ hybridization;
MI: Metaphase I;
ML: Maximum likelihood;
PCR: Polymerase chain reaction;
PP: Posterior probability
