# Supplementary material for: Biosystematics studies on Elymus breviaristatus and Elymus sinosubmuticus (Poaceae: Triticeae)
Source: BMC Plant Biol. 2022 Feb 1;22:57. doi: 10.1186/s12870-022-03441-y (PMC8805286; doi:10.1186/s12870-022-03441-y)
Supplement: Supplementary file 1 — Additional file 1. [file 12870_2022_3441_MOESM1_ESM.pdf]

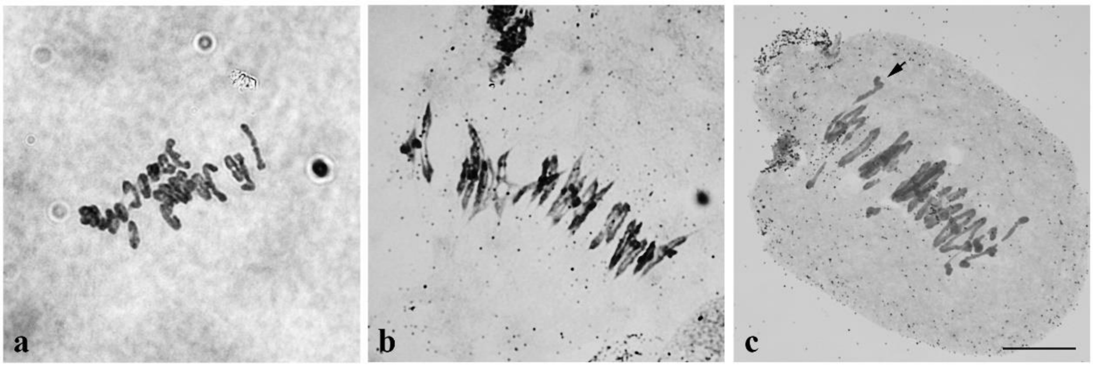

Fig. S1 Meiotic chromosome pairing at metaphase I in parents and hybrids. **a**, *E. breviaristatus* (**StYH**) with 21II. **b**, *E. sinosubmuticus* (**StYH**) with 21II. **c**, *E. breviaristatus* × *E. sinosubmuticus*, 19II + 1IV(arrows). Scale bar equal 10 μm.
